# The Atomic Circus: Evidence-Based Chemistry Demonstration Theater

**DOI:** 10.1021/acs.jchemed.5c01050

**Published:** 2026-02-24

**Authors:** Matt Queen, Amanda Obery, Ted M. Clark, Martha Cabell, Shelly Hogan

**Affiliations:** † 33049 Montana State University Billings, Billings, Montana 59101, United States; ‡ 3197 Central Washington University, Ellensburg, Washington 98926, United States; § 2647 The Ohio State University, Columbus, Ohio 43210, United States; ∥ Yellowstone Evaluation Services, Bozeman, Montana 59715, United States

**Keywords:** Elementary Science, Demonstration Theater, Models, Phases, Reactions, Kinetic-Molecular Theory

## Abstract

*The Atomic Circus* is a research-based chemistry demonstration theater designed to engage elementary audiences and their families through narrative, music, and dance. Guided by design principles of age-appropriate conceptual framing, narrative storytelling, and theatrical metaphor, the show integrates live demonstrations within a three-act performance that follows a “Novice” character who learns alongside the audience. A mixed-methods evaluation, including pre- and postshow surveys and family interviews, revealed strong affective engagement and knowledge gains around physical and chemical change. These outcomes reflect the broader trends identified in recent reviews of science shows, where enthusiasm and modest knowledge gains are consistently observed, but systematic documentation remains limited. By transparently documenting its design, logistics, and evaluation, *The Atomic Circus* contributes to the small but growing evidence base on science theater and encourages other practitioners to share systematic accounts that connect creative performance with educational theory.

## Graphical abstract

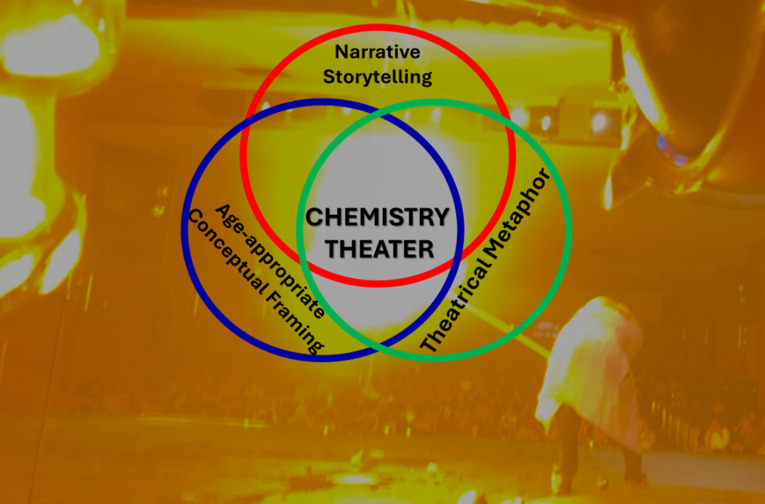

Chemistry demonstration shows capture audiences’ attention and emotion, and have been a part of teaching and learning in the field since their inception.^1^ Chemistry demonstration shows have been a fundamental part of outreach efforts in large part due to their wide accessibility. Early examples, especially those from the 1970s and 1980s, established a durable repertoire of demonstrations that circulated widely across outreach contexts.^1–5^ These shows captured attention through spectacle, popularized chemistry for lay audiences, and emphasized the excitement and safety of chemical experimentation. Many of their demonstrations remain familiar today, reflecting the long-standing appeal of this model.

Building on this tradition, a more recent work has emphasized pedagogical framing. Some shows are now described as “demonstration theater,” with experiments staged as coherent performances. Initiatives such as the Fusion Science Theater program^6,7^ and the Atom Surprise^8^ incorporate humor and weave in an overarching narrative. Such approaches transform chemistry demonstrations into stories that give purpose, sparking curiosity and excitement. These examples highlight the creativity of practitioners who frame demonstrations not simply as spectacles but as vehicles for meaning-making and sustained engagement.

Scholarship has followed suit, examining demonstration shows with increasing theoretical and methodological rigor. 9445Work drawing on drama and storytelling^6–8^ emphasizes that demonstrations can be strengthened when presented as narratives rather than isolated tricks, engaging audiences through structure and meaning. The development of the Fusion Story Form^10^ exemplifies this hybrid approach, embedding conceptual explanations directly within narrative arcs. Advances in assessment have also enabled researchers to probe children’s conceptual understanding in live performance contexts, with new tools^9^ offering insights into how chemical ideas are grasped during demonstrations. This orientation reflects the growing recognition that effective science communication can enhance cognitive clarity through the theatrical, emotional, and structural power of stories.

Duggan and colleagues’ recent systematic review situates science shows within this evolving landscape.^11^ They describe the genre as a versatile hybrid, borrowing elements from lectures, theater, videos, and even magic shows yet retaining a distinct identity as live performance. Across this diversity, one outcome consistently emerges: science shows engage audiences. Enjoyment, curiosity, and enthusiasm are frequently reported alongside occasional cognitive gains, making engagement the defining hallmark of the genre. At the same time, Duggan and colleagues^11^ underscore how limited the evidence base remains. Only a handful of peer-reviewed studies in the past decade have met their criteria, underscoring the scarcity of systematic reporting and the difficulty of drawing conclusions across such heterogeneous contexts.

This scarcity of articles highlights the importance of detailed, systematic reports of activity. Each well-documented example contributes to a cumulative understanding of science shows, illustrating how they are conceived, implemented, and evaluated in practice. *The Atomic Circus*, as shared in this article, can be understood in this light: not as a definitive model but as one contribution to a larger collective effort to strengthen the evidence base. By situating its design choices, guiding design principles, and evaluation strategies within the categories identified in Duggan’s^11^ review, this case study offers transparency and comparability, helping to expand the small but growing set of systematic accounts that can inform future synthesis.

## Setting the Stage for Demonstration Theater

Duggan and colleagues^11^ proposed a set of categories—aims, logistics, evaluation, and outcomes—that provide a structure for comparing across otherwise heterogeneous contexts. Their analysis revealed that science shows are typically designed around dual aims: promoting affective outcomes such as curiosity, excitement, and enjoyment and fostering cognitive outcomes tied to knowledge and conceptual understanding. These dual aims echo findings across the wider informal science education literature, where affective engagement is consistently emphasized as central to learning experiences.^12,13^ Other reviews have suggested an expanded set of aims, with Austin and Sullivan^14^ including behavioral dimensions such as encouraging follow-up activities and motivating continued participation in science. Some studies have also highlighted identity-related aims, such as enhancing science self-efficacy or broadening students’ perceptions of who can be a scientist.^15^ Yet across this literature, aims are not always clearly articulated and affective goals tend to be emphasized far more consistently than cognitive, behavioral, or identity-oriented ones. This unevenness underscores a central challenge for science shows: whether their purpose is primarily to entertain, educate, inspire, or cultivate longer-term engagement with science.

In addition to the aims, the review highlights the logistical diversity of science shows, which complicates efforts to compare across cases. Science shows have been staged in formats ranging from brief demonstrations, traveling academic talks,^16,17^ to hour-long performances, and they occur in a wide variety of settings including science centers, museums, festivals, schools, and community venues. Presenters are equally diverse—scientists, educators, communicators, and professional performers have all taken on the role of delivering shows. This variety illustrates the adaptability of the format but also underscores why systematic study is difficult: factors such as length, venue, audience demographics, and presenter identity can significantly shape outcomes. Similar challenges have been documented in broader informal science education research, where exhibit and program design must account for differences in context and audience.^18^ Chemistry outreach, specifically, also shows this diversity, as highlighted in Holme’s recent retrospective in the *Journal of Chemical Education*, which traces efforts ranging from chemistry “magic shows” to museum partnerships and summer camps, each adapted to different audiences and institutional purposes.^19^ Other studies reinforce how design choices influence engagement: Roche et al. emphasize the role of practicing scientists and interactive technology,^20^ Phillips et al. point to humor and respectful interaction as key for teenage audiences,^21^ and Howell et al. highlight the impact of near-peer role models for younger students.^15^ Together, these perspectives reinforce Duggan’s point: without consistent documentation of logistical details, it is difficult to compare results across studies or to build a cumulative understanding of how context shapes outcomes.^11^

Evaluation emerged as another central theme. Science shows have been assessed using a wide range of methods, from surveys and interviews to observational studies, yet there is little consistency in how outcomes are measured. Reported findings span both affective outcomes—such as enjoyment, curiosity, and positive attitudes toward science—and cognitive outcomes, including gains in knowledge and understanding. Across this variation, one result stands out: audience engagement emerges as the most consistently observed impact regardless of format, setting, or audience type. Similar challenges have been highlighted in the wider informal science education field, where evaluations often struggle to capture both immediate affective responses and longer-term cognitive impacts.^22^ To improve practice, resources such as *The Practical Evaluation Guide* ^23^ offer tools for aligning evaluation strategies with program goals and contexts. Recent studies also demonstrate alternative approaches: short, open-ended interviews administered immediately after demonstrations can capture children’s reasoning in authentic informal settings,^9^ while audio-recordings of unstructured postshow conversations provide insights into spontaneous curiosity and sense-making.^24^ A small number of studies even extend evaluation longitudinally, with evidence that memorable demonstrations can be recalled and applied years later.^25^ Yet despite these innovations, broader critiques of science communication evaluation remain relevant: assessments are often methodologically weak, under-theorized, or framed primarily as success stories rather than genuine learning opportunities.^26^ Taken together, these perspectives reinforce Duggan’s conclusion: without intentional and standardized approaches, it remains difficult to compare results across studies or to build a coherent evidence base for the impacts of science shows.^11^

Finally, the advancement of science shows research depends on more consistent reporting practices. Future studies must clearly articulate their aims, describe logistical details, and align evaluation strategies with these contexts. Establishing such standards would not only enable more meaningful comparisons across studies but also help build a stronger cumulative evidence base for the field. Similar calls for systematic reporting have been made across informal science education more broadly,^13^ underscoring that science shows face not only the challenge of evaluation but also of consistent documentation. It is in this spirit that the present article provides a transparent account of the aims, design, and evaluation of *The Atomic Circus*, contributing to the small but growing set of systematically reported science shows.

## The Atomic Circus

Chemistry theater productions developed by *The Atomic Circus* fit within Duggan et al.’s call for transparency as contributions to the limited evidence base.^11^ The shows were designed through an evidence-based approach that views science engagement as both dynamic and affective. While not developed in response to Duggan’s review, the productions were shaped by an articulated set of design principles that connect performance choices to research on learning and engagement.^11^ In this way, the shows illustrate how educational research can inform practice while also extending the comparability of performance-based outreach. *The Atomic Circus* fifth grade show case study presented here is not a universal model, but instead, is one example that others may adapt when developing theatrical strategies for informal chemistry education. To ensure transparency, a fuller descriptive account of staging and narrative structure is included in the Supporting Information.

## Research-Informed Design Principles

Where many science shows rely on intuition or tradition, the structure of *The Atomic Circus* shows was guided by a set of three research-informed design principles. First, age-appropriate conceptual framing ensures that content aligns with the developmental stage of the target audience, connecting abstract ideas like particle motion to tangible experiences.^29–31^ Second, narrative storytelling uses character arcs and emotional investment to scaffold cognitive engagement and provide continuity between demonstrations. The approach builds on evidence that dramatized stories foster both affective and cognitive learning.^32^ Finally, theatrical metaphor leverages music, dance, and visual symbolism to help audiences reason about unseen scientific phenomena, resonating with literature on embodied and multimodal learning.^36–38^ Together, these design principles work in concert to support both emotional resonance and conceptual sense-making.

These design principles serve as a flexible framework that shapes choices about narrative, audience engagement, conceptual depth, and logistical form. Importantly, the principles are applied across all *Atomic Circus* shows, but the diverse aims of each production lead to different expressions of the principles through varied practices. There are some principle-informed practices found in all *Atomic Circus* shows. A general template for designing an *Atomic Circu*s show can be found in the Supporting Information. The fifth-grade production described here was iteratively developed over multiple years, grounded in research across science education, narrative learning, and performance studies.^27,28^ Across all *Atomic Circus* productions, the guiding through-line is the creation of entertaining, educational theater that transforms scientific concepts into a lived experience.

Although *the Atomic Circus* differs in scale and artistic scope from Fusion Science Theater, it shares several key elements of the Fusion Story Form (FSF). These include the use of a guiding scientific question, structured prediction–observation cycles, character roles that scaffold sense-making, and a narrative resolution that articulates the target concept. *The Atomic Circus* expands these components through dance, music, and large-scale demonstrations, but the underlying pedagogical architecture is consistent with FSF principles. Table S4 summarizes these points of alignment and distinction.

1 **Synopsis of The Atomic Circus Fifth Grade Show**

In *The Atomic Circus*, a college student is “tricked” into joining a chemistry class and taking on the part of the Novice, a surrogate for the audience’s own learning. Each time the Novice encounters a spectacular demonstration, the Expert—a humorous scientist—offers an explanation, modeled with building bricks, that lulls the Novice to sleep, transporting the show into the fantastical Atomic-Level Circus where dancers embody more sophisticated moving molecular models of the process. Awakening with new insight, the Novice revises their understanding and makes predictions about ever intensifying demonstrations, from melting ice to imploding steel barrels and explosive chemical reactions. Supporting characters function as archetypes: the Safety Officer is stern and orderly, emphasizing rigor and precaution, while the Lab Technician represents the calculated urge to push the limits. Together, this blend of demonstrations, narrative, live music, and dance transforms physical and chemical change (NGSS 5-PS1–4)^31^ into an immersive theatrical arc where the Novice’s growth mirrors the audience’s own journey from curiosity to conceptual clarity.

## Program Aims and Rationale

Duggan and colleagues’ review emphasized that aims are often the least clearly articulated aspect of science shows, even though they are central to understanding affective and cognitive outcomes.^11^ They also observed that affective goals are consistently highlighted, while cognitive, identity, and broader community domain aims appear far less often. *The Atomic Circus* was deliberately designed with articulated goals across all four domains. These aims are guided by the evidence-based design principles outlined above, linking performance choices to research in informal science education, narrative learning, and chemistry education.

The first aim is to foster positive affective dispositions toward science, emphasizing curiosity, enjoyment, and relevance (Figure 1). Research in informal contexts consistently highlights affective engagement as central to learning experiences, where enjoyment and emotional investment provide a foundation for sustained interest in science.^12,33,34^

**Figure 1:**
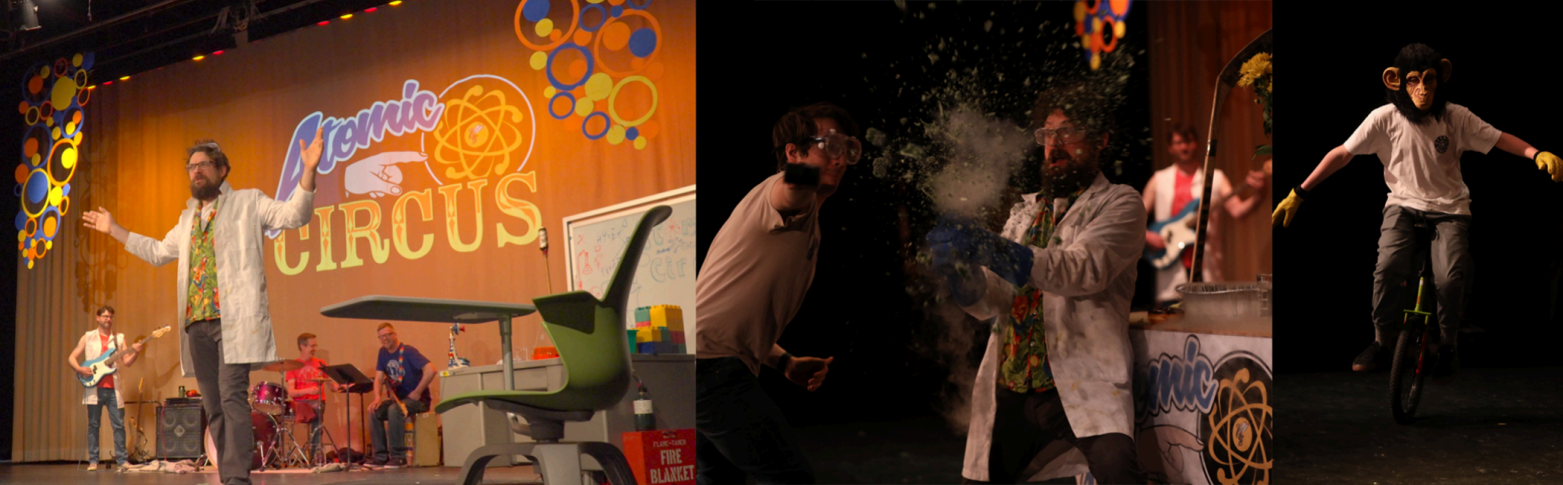
*The Atomic Circus* uses spectacles to foster positive affective dispositions toward science.

A second aim is to strengthen conceptual insight into disciplinary core ideas aligned with the age-appropriate Next Generation Science Standards (NGSS),^31^ particularly the fifth-grade standard 5-PS1-4 on physical and chemical change. This reflects a broader principle of developmental alignment, which emphasizes that science interventions must be designed with learners’ cognitive readiness in mind.^29–31^ Such aims echo the call to ground chemistry education in learning progressions and model-based reasoning.^28,39^

Third, *The Atomic Circus* seeks to broaden students’ perceptions of science and scientists by situating the performance in a university theater while representing scientists and learners in diverse and relatable ways. Prior studies highlight the importance of identity, belonging, and representation in shaping science attitudes.^15,40,41^ Therefore, *The Atomic Circus* aimed not only to expose elementary students to higher education environments but also to offer positive, humanized models of scientific practice through characters such as the Novice, Expert, and Safety Officer.

Finally, the project aimed to engage families and communities through shared cultural experiences of science theater (Figure 2). Place-based and community-integrated approaches to STEM education emphasize the value of situating science within familiar contexts and leveraging local resources.^42–44^ By incorporating public family shows and collaborating with local dancers and musicians, *The Atomic Circus* was designed to treat science as both a communal and an educational endeavor.

**Figure 2:**
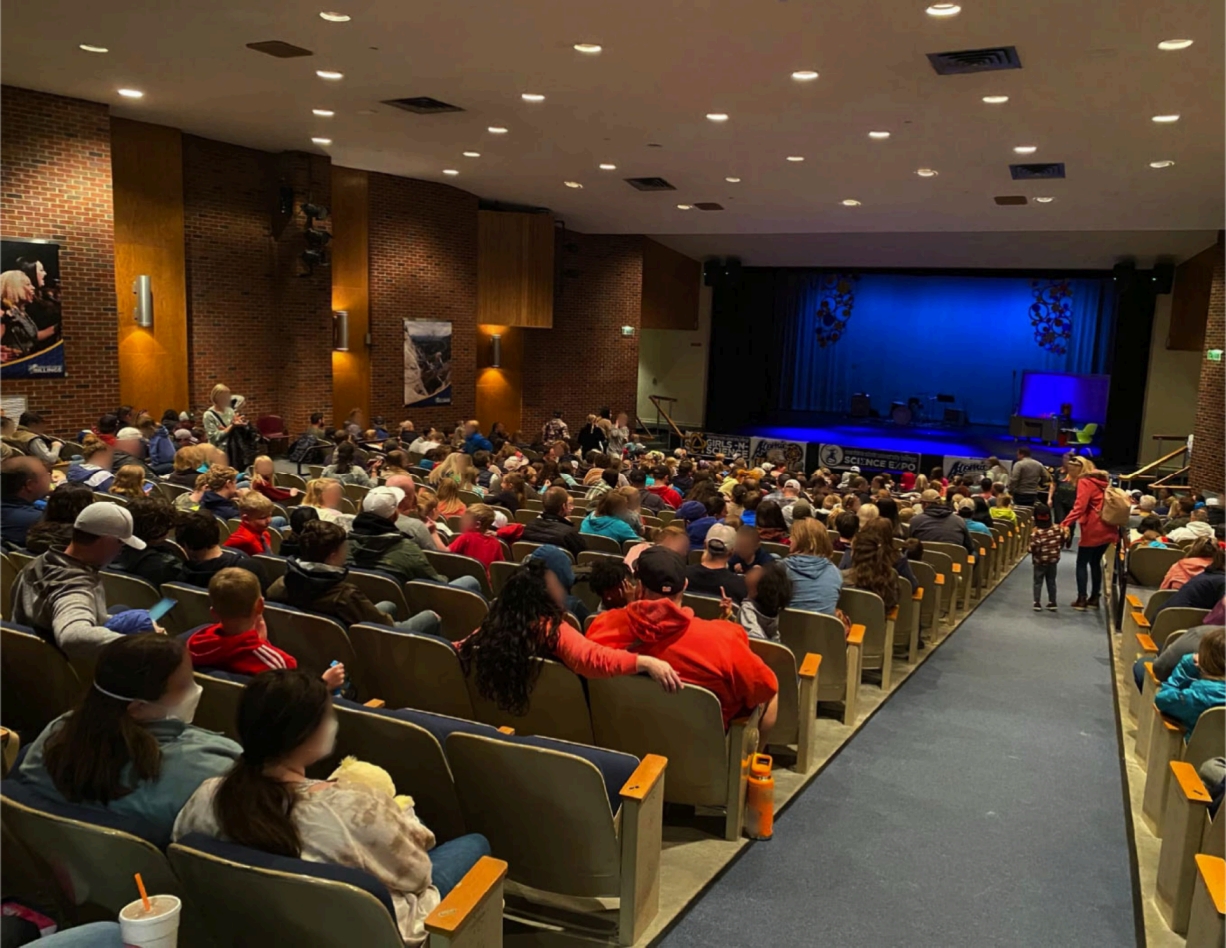
In addition to hosting two days of fifth-grade field trips, *The Atomic Circus* hosts a free public night annually using theatrical elements including lights, music, and aesthetic narrative components to give the audience a sense of setting and purpose.

Together, these aims reflect an intentional effort to balance affective, cognitive, identity, and community goals rather than relying on spectacle alone. While many of the reviewed science shows emphasized conceptual understanding, often complemented by affective aims, fewer incorporated identity or community engagement. By deliberately spanning all four domains, *The Atomic Circus* addresses the imbalance noted by Duggan and colleagues and contributes a broader model of what science shows can aim to achieve.^11^

## Logistical Structure and Narrative

Duggan and colleagues emphasize that outcomes of science shows are inseparable from the conditions under which they are staged—factors such as duration, venue, and presenter identity all shape the nature of the show. Yet these details are often underreported, making it difficult to compare across cases.^11^ To address this gap, a transparent account of the logistical features of *The Atomic Circus*, linking to the design principles that shaped its design, is shared. A summary table in the Supporting Information extends Duggan’s published comparisons with an additional column for *The Atomic Circus*, enabling direct alignment with prior documented shows.^11^

Since its inception, the *Atomic Circus* fifth-grade production has been performed over 50 times for approximately 16,000 students across Montana. The full theatrical production incorporates elements such as video vignettes, artistic animated models, live musical stings, tightly choreographed dance pieces, and comedic improv beats that maintain momentum between demonstrations. These theatrical elements allow the narrative to flow continuously, despite the variable timing inherent in large-scale chemical demonstrations. As a result, the pacing remains energetic and coherent as the plot unfolds. Each full performance runs for approximately one h and follows a narrative through a three-act structure. The first act introduces the Novice, a college student drawn into a chemistry “class,” whose role is to serve as a surrogate learner for the audience (Video S2). Anchoring the show in a character who learns alongside viewers reflects the principle of narrative storytelling, which positions identification with a character as a scaffold for cognitive engagement.^32,33,35^ The second act introduces the Novice to physical change, with demonstrations such as ice race, balloon immersion in liquid nitrogen, and a steel barrel implosion. These demonstrations are deliberately staged as cycles of prediction, observation, and revision, aligning with cognitive science research that emphasizes how understanding develops through iterative reasoning processes (Figure 3).^39,45^ Importantly, these cycles are not isolated but woven into a narrative arc, reflecting Fisch’s capacity model, which highlights that content and storyline must be tightly integrated for audiences to process both effectively.^8,45^

**Figure 3:**
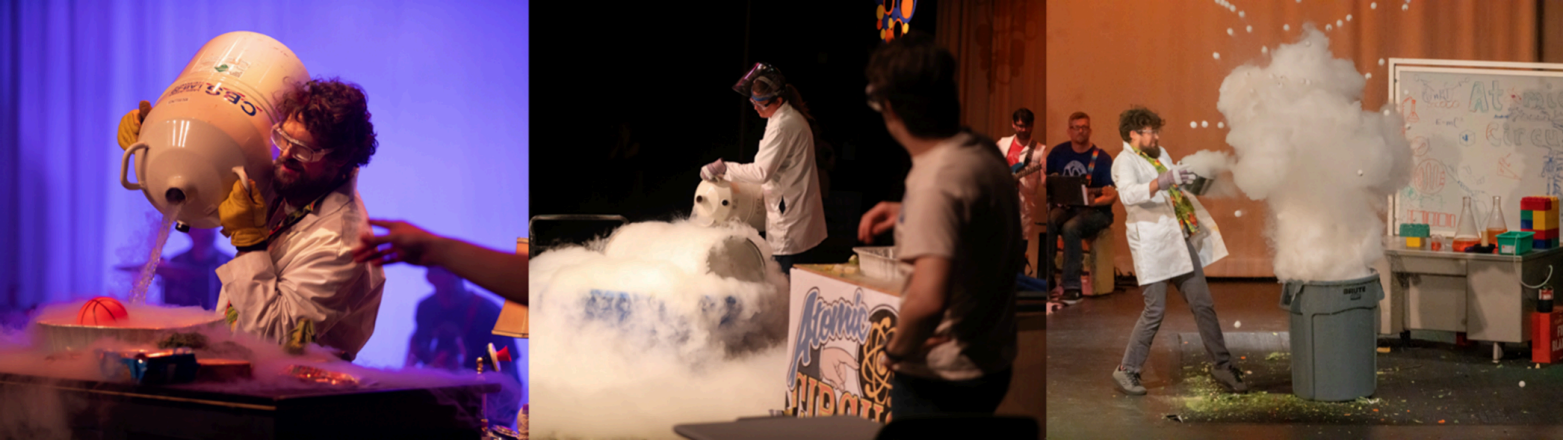
Scaled demonstrations create cycles of prediction where liquid nitrogen is first used to cool a basketball, allowing for the atmosphere to crush it, using liquid nitrogen to cool a 50-gallon drum, allowing for the atmosphere to implode it, and then using hot water to heat liquid nitrogen, vaporizing it and creating an explosion of vapor. Each sequence follows a Predict–Observe–Explain learning cycle that parallels how models are tested and refined in science.

During the show, the Expert often introduces concepts with building blocks as a physical model for atoms, discrete pieces that can combine, separate, and be rearranged. While this model helps the novice visualize matter’s particulate nature, it is limited in representing energy and motion. Later, the interpretive dancers expand the model’s scope: their movements translate thermal energy into rhythm and spacing, allowing the audience to feel the kinetic molecular theory through embodied motion. The use of the two models illustrates how scientists shift between representations depending on the questions they ask (Figure 4). It is discussed how each model has distinct affordances (clarity and movement) and limitations (static structure, scale). Discussing these trade-offs reinforces modeling as a process of continual refinement rather than a quest for a single perfect depiction.

**Figure 4:**
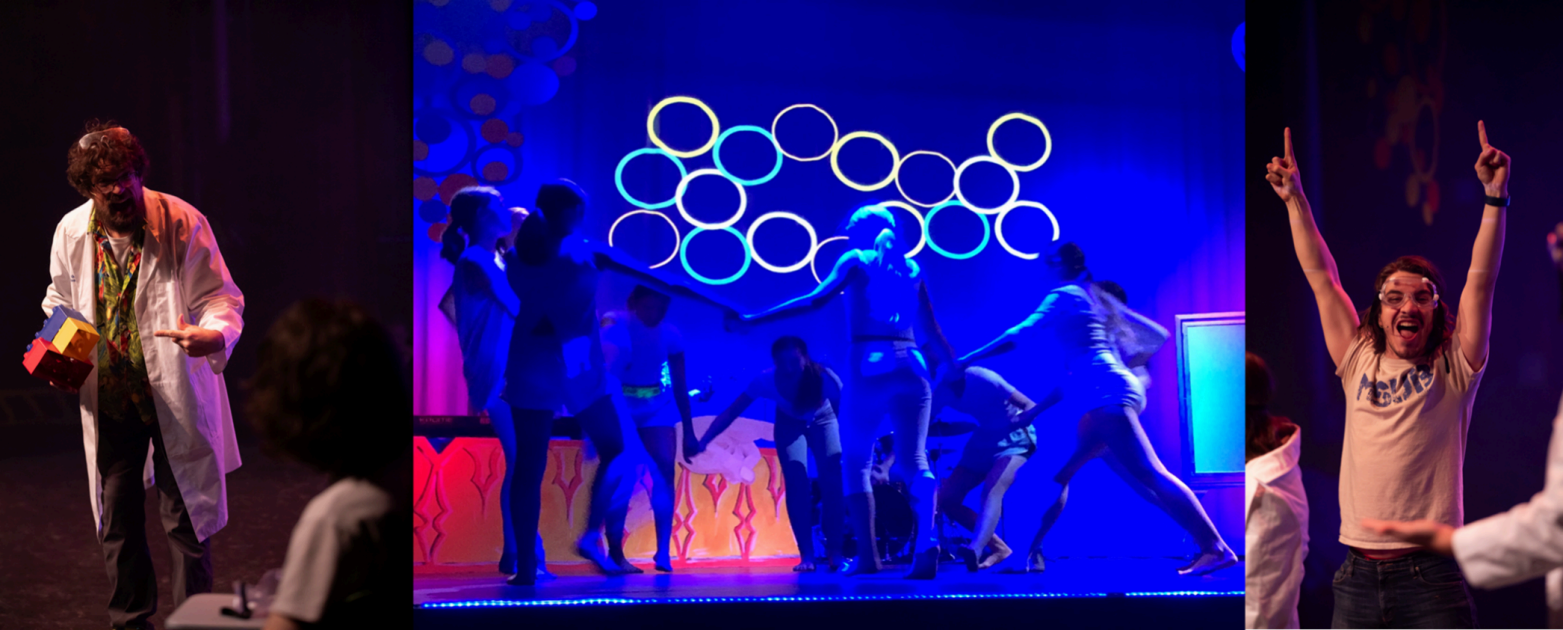
Expert (chemistry professor) explains the concepts using a physical model, and dancers (local dance troupe) provide a conceptual model showing how molecules move during a chemical or physical process, and the audience feels invested in the success of the novice (college student actor).

The final act culminates in a series of chemical change demonstrations incorporating combustion, oxidation, and synthesis reactions, dramatized with music and dance, that underscore the Novice’s transformation from a lack of understanding to conceptual clarity. These scenes explicitly address the NGSS 5-PS1–4 ^31^ performance expectation, highlighting how mixing or reacting substances can produce new substances with distinct properties. This resolution exemplifies the principle of age-appropriate conceptual framing, in which disciplinary core ideas are presented at a level consistent with the NGSS fifth-grade standards.^29–31^

Performances are staged in university theaters equipped with sound, lights, and projection. Staging is deliberately minimal—anchored by a professor’s desk, a rolling student desk, a whiteboard, and a mobile cart that brings demonstrations on and off stage (full technical details can be found in the Supporting Information). This restraint reflects the principle of theatrical metaphor: keeping physical staging simple makes space for interpretive dance, live music, and projection to envision otherwise invisible processes^36–38^ allowing symbolic and embodied elements to remain the audience’s primary cognitive anchor. Although more modern community theaters were available, the university venue was selected for symbolic reasons: for many fifth-grade students, attending a performance in a college theater was their first encounter with higher education. Prior work shows that exposure to such environments can help shape identity and belonging in science, particularly for students from underrepresented backgrounds.^15,40,41^

The ensemble of presenters further illustrates how the show integrates scientific authority with theatrical and artistic expertise (Video S3). Two chemistry faculty members play the role of Expert and Safety Officer, modeling both scientific credibility and safe practices (full safety and risk management plans can be found in the Supporting Information). Two college-aged actors are cast in the Novice and Lab Technician roles to ensure strong character work, comedic timing, and improvisational ability. Because demonstrations sometimes take longer or shorter than expected, the actors’ improvisational training ensures that transitions remain seamless, maintaining audience attention and stabilizing pacing throughout the show. A three-piece rock band provides live scoring, improvising in the style of a late-night show band to accent dramatic beats; in some communities, school bands have filled this role, reinforcing local participation. Finally, a troupe of modern youth dancers from local studios embodies molecular-level phenomena through choreographed movement. This cross-disciplinary collaboration demonstrates what Chemi and Kastberg describe as the “typologies of science theater,” in which scientists, actors, and artists create hybrid performances that communicate science through multiple modalities (Figure 4).^36^

In terms of logistics, *The Atomic Circus* both reflects and diverges from patterns reported in Duggan and colleagues’ review.^11^ Like other shows, it employs a bounded, compact time frame and adapts staging to local resources, but at roughly one h it is notably longer than most reviewed examples, some of which ran only 20–25 min. This extended format enables a full three-act narrative, allowing affective, conceptual, and identity-related elements to develop in tandem. The show also shares features with earlier studies in its hybrid use of scientists and performers while extending this practice by incorporating live musicians and youth dancers to embody molecular processes. Similarly, the choice of a university theater contrasts with festivals, schools, and science centers featured in prior work, situating young audiences in a symbolic higher-education environment. Taken together, these similarities and differences highlight how *The Atomic Circus* exemplifies common practices in science shows while also extending their logistical scope.

## Science Show Evaluation

Evaluation has long been a feature of science shows, but its purposes and audiences have varied. As Duggan and colleagues observe, most evaluations were historically conducted for stakeholders such as funders, program managers, or participating institutions, rather than for academic readership.^11^ They often emphasized documenting reach, satisfaction, or immediate impact over building a cumulative research evidence base. The exploratory evaluation of *The Atomic Circus* fits within this tradition: it was designed to assess outcomes of interest to program stakeholders while also providing transparency about design and learning about the audience experience. Sharing the approach here aims both to honor that context and to extend the broader conversation Duggan initiated, highlighting how pragmatic program evaluation can nonetheless contribute to science education research.^11^

Evidence of the potential of the *Atomic Circus* framework comes from a mixed-methods design that combined a pre- (15 Likert and 1 open-ended question) and postshow (16 Likert and 1 open-ended question) survey with postshow interviews during a public performance targeting upper elementary students; see Supporting Information. The surveys, approved by the Institutional Review Board from Montana State University Billings (IRB00001622) and distributed via QR codes in the theater lobby, assessed participants’ attitudes toward science, enjoyment of the show, and learning of key science concepts. Interviews, conducted immediately after the show, targeted families with upper-elementary-aged children (18 questions, see Supporting Information). This dual survey-and-interview approach parallels strategies used in earlier science show evaluations. For comparison, Fish et al. used multiple-choice pre/posttests with school groups;^46^ DeKorver et al. relied on brief semistructured interviews;^9^ Roche et al. emphasized real-time audience polling;^20^ and Karim & Roslan combined structured testing with follow-up interviews.^47^ By contrast, Stojanovski^48^ reported no formal evaluation at all. Within this varied landscape, *The Atomic Circus* represents an exploratory approach; one that aims to gain a holistic sense of the audience experience, by using multiple measures exploring a number of constructs while honoring the informal nature of the experience.

Consistent with informal contexts, the survey response rates were low and presented clear limitations to the data. A total of 66 unique responses were collected (n = 21 preshow; n = 54 postshow). The audience demographic was predominantly white (76%) and highly educated (83% with postsecondary experience), with many attendees bringing children. Among minors, the median age was 8. Postshow interviews added further depth, with 14 interviews conducted across 49 individuals (21 adults and 28 minors). In scale and scope, these numbers align with other science evaluations, providing a reasonable point of comparison for outcomes.

### Affective Evaluation

Presurvey items share about the families attending the event and their level of engagement with science prior to attending *The Atomic Circus*; see Table 1.

**Table 1:** Pre-survey Questions

Question	Mean^a^	Standard Deviation
We are interested in science	4.43	0.96
We discuss science at home	3.95	1.55
We seek out out-of-school opportunities to learn science	4.10	1.39
Learning about science is important to our family	4.24	1.39
Science is part of our daily lives	4.43	0.86
We enjoy learning about science in school	4.38	1.05
Our family works or aspires to use science in their careers	4.10	0.89

a Likert scale items with responses of 5 being “Strongly Agree.”

These affective items were single-item measures rather than multi-item subscales, a common approach in informal science evaluations where instruments must remain brief. Participants reported levels of enjoyment and interest in the performance in the postsurvey, see Table 2.

**Table 2:** Post-survey Questions

Question	Mean^a^	Standard Deviation
My family enjoyed the ACES Show	4.92	0.28
This kind of out-of-school opportunity is important for increasing interest in science	4.98	0.14
The ACES Show related to my community	4.71	0.61
I would attend a similar event in the future (i.e., family science event)	4.94	0.23
The Show was well-produced	4.94	0.23

a Likert scale items with responses of 5 being “Strongly Agree.”

Open-ended survey responses to the question, “*Why did you attend the show today*?” emphasized the value of entertainment for sparking learning with responses such as, *“The kids and explosions are awesome! And it is such a good learning event,” and, “to learn more about chemistry and see some experiments.”*

Interviews highlighted how multimodal elements shaped engagement; see Supporting Information for the interview protocol. Parents and children pointed to dancers, music, lights, and humor as central to making science concepts memorable. A parent remarked, *“I really loved the aspect of the dancers ... that was really good at showing how the molecules move in such a different way.”* Even a four-year-old noted the mix of sensory experiences: *“some parts were loud and some parts were quiet and some parts were colorful and some parts were cool.”* Such accounts help provide evidence toward the impact of how narrative structure and theatrical metaphor enhancing affective engagement. These results mirror the affective outcomes most consistently reported in prior science show studies,^11^ and support show development by linking specific design choices—such as the Novice character and the integration of music and dance—to audience experience.

### Cognitive Evaluation

Survey data showed evidence of learning core concepts, including molecular motion and the distinction between physical and chemical change; however, these are limited and exploratory in nature as open-ended questions and interviews did not ask participants to describe a particular demonstration or concept. However, confidence in identifying physical versus chemical change increased significantly from pre- to postshow as seen in a Welch’s *t* test (M_pre = 3.57, SD = 0.75; M_post = 4.34, SD = 0.92; t(72) = 3.41, p = 0.001; *d* = 0.88). Correct responses on other targeted items also improved, such as recognizing phase changes as physical (81% → 89%) and comparing molecular motion in gases vs liquids (76% → 94%). Brief postsurvey comments reinforced this pattern (e.g., *“Learned the difference between chemical and physical changes”*). Interviews provided richer evidence, with one-fifth-grader noting, *“If you heat things up and give it more energy, then things will move faster. And then if you make it much colder, the energy will be gone and it will be slow.”* (Table 3).

**Table 3:** Pre- and Post-survey Percentage of Correct Responses to Knowledge Questions

Question	Correct Pre (*n* = 21)	Correct Post (*n* = 54)
When you put two substances together and a new substance is created, that is a _______ change.	86%	93%
When substances change their state (i.e., gas to liquid, or liquid to solid) this is a _______ change.	81%	89%
When substances are heated, do their molecules move faster or slower?	86%	96%
Do molecules move faster in a gas than they do in a liquid?	76%	94%

An open-ended question in the postsurvey asked participants to share what they learned at the show. Responses were brief and unspecific, but highlighted the content focus of the show with quotes such as “*Learned the difference between chemical and physical changes*,” “*Cause and effect of liquid nitrogen*,” and “*How molecules change states at different temperatures*.”

Interviews shared a greater depth about cognitive outcomes, although they are not without limitations with multiple family members being present and the short duration of the interviews. A fifth grade student spoke of physical and chemical change when he said, “*... if you heat things up and give it more energy, then things will move faster. And then if you make it much colder, the energy will be gone and it will be slow*.” A student, aged 9, highlighted modeling, sharing, “*the legos and modeling the elements ... in my science class, my teacher had done that.”* Hearing how audience members grapple with core content and make connections to their everyday lives helps to inform future iterations of how the show frames content within demonstrations and explanations.

Taken altogether, these findings echo a recurrent theme in the literature: affective outcomes are consistently strong, while evidence for content gains is less reliable. Many prior studies relied on general self-report items (*“I learned something new”*) or very brief interviews, limiting claims about conceptual change. In this mixed landscape, *The Atomic Circus* offers a modest but noteworthy contribution: by combining survey and interview data that probe family engagement and learning while providing feedback to the show for future iteration.

## Limitations and Future Directions

Although the results from *The Atomic Circus* are encouraging, several limitations constrain interpretation. First, the evaluation was designed with program stakeholders in mind rather than as a formal chemical education research study. This, along with the informal context, shaped the brevity of survey instruments, the modest sample size, the self-report nature of the instruments, and the reliance on short, immediate postshow interviews with multiple family members. This evaluative approach is both a limitation and supportive of a goal to improve production in that questions asked through the tools were often broad (see Supporting Information), enabling participants to share openly but causing issues with reliability and validity. Second, the findings reflect a single production in one cultural context, that is, an urban Montana community, limiting generalizability. Third, the audience was predominantly white and highly educated, underscoring the need to test the approach in more diverse settings. Finally, data were collected immediately after shows through surveys and short interviews, which capture audience impressions but not long-term impact or conceptual change. These limitations are typical of early stage outreach evaluations but highlight the need for longitudinal and classroom-embedded follow-up studies that connect audience experiences to student learning outcomes.

Future research could build on this foundation by adopting richer interview protocols,^9^ incorporating real-time audience polling,^20^ exploring alternative assessment formats such as drawings, or conducting longitudinal follow-ups to assess persistence of outcomes. Comparative studies across multiple shows, themes, and communities also provide stronger evidence of transferability. By documenting aims, design choices, and evaluation results in detail, *The Atomic Circus* contributes to the small but growing set of systematically reported science shows and helps chart directions for a more cumulative evidence base in informal chemistry education.

## Conclusions

This study of *The Atomic Circus* contributes to the small but growing body of systematically reported science shows by offering a transparent account of its design principles, aims, logistics, and evaluation. While not intended as a universal model, it illustrates how evidence-based choices—age-appropriate conceptual framing, narrative storytelling, and theatrical metaphor—can be enacted in practice and connected to outcomes that span affective, cognitive, identity, and community domains. In doing so, it responds directly to the call by Duggan and colleagues for more detailed accounts that make science shows comparable across contexts and evaluable within a broader research conversation.^11^

Even with limitations, this study underscores the importance of documentation itself. Even when evaluations are modest in scale and oriented toward program stakeholders, sharing accounts adds to the sparse data set that others can learn from and build upon. Practitioners and researchers alike should be encouraged to publish their experiences—whether large-scale or small, innovative or traditional—so that a fuller picture of science shows practice can emerge. Future work must occur, as progress depends on sustained contributions of descriptive accounts alongside methodological refinement. More broadly, *The Atomic Circus* illustrates how creative performance, where curiosity, narrative, and spectacle combine, can make chemistry feel alive and accessible. The heart of the project lies not in proving a single model but in demonstrating that well-crafted theater can cultivate scientific thinking and wonder in equal measure.

By valuing transparency and encouraging collective participation, the science-education community can continue to strengthen the evidence base while preserving the imaginative energy that makes science shows a distinctive and enduring form of engagement.

## Supplementary Material





## Notes

Detailed stage design and technical setup; full narrative synopsis of *The Atomic Circus*; comparison table aligning the production with studies from Duggan et al.; mapping of evidence-based design principles to show elements; extension of the Fusion Story Form; general template for future productions; and safety and risk management procedures (PDF, DOCX)
