# Supplementary material for: The Atomic Circus: Evidence-Based Chemistry Demonstration Theater
Source: J Chem Educ. 2026 Feb 24;103(3):1346–54. doi: 10.1021/acs.jchemed.5c01050 (PMC12980712; doi:10.1021/acs.jchemed.5c01050)
Supplement: Supplementary file 1 [file ed5c01050_si_001.pdf]

Supporting Information

The Atomic Circus: Evidence-Based Chemistry Demonstration Theater
Matt Queen*, Amanda Obery**, Ted M Clark***, Martha Cabell****, and Shelly Hogan****

*Montana State University Billings, Billings, Montana, 59101, United States
**Central Washington University, Ellensburg, Washington, 98926, United States
***Ohio State University, Columbus, OH, 43210, United States
***Yellowstone Evaluation Services, Bozeman, Montana, 59715, United States

Table of Contents

Page S1. Atomic Circus Template

Page S3. Safety and Risk Management.

Page S4. Stage Design and Technical Setup.

Page S5. Full Show Narrative Overview.

Page S9. Evidence-Based Design Principle mapped to show.

Page S10. Incorporation into Duggan et al.'s review chart.

Page S11. Extension of the Fusion Story Form.

Page S12. Evaluation Tools.

Page S14. References

Atomic Circus Template

Across productions, the creative process followed a consistent design template rooted in the three framework principles. Each show begins by defining a single core concept aligned with NGSS performance expectations and mapping it to a three-act narrative arc that mirrors a learning cycle: (1) an initial encounter with a puzzling phenomenon, (2) exploration and model building through scaled demonstrations and discussion of model limitations and affordances, and (3) resolution through synthesis and application. The principles of age-appropriate framing, narrative storytelling, and theatrical metaphor guide decisions about language level, character interactions, and demonstration sequencing.

All shows include the core, Novice, Expert, and Safety Officer characters interacting in a three act format. The first act is centered around introducing the Novice and allowing them to encounter the puzzling phenomena (Figure S1). The next act involves building up the models from simple physical models to more complex theatrical metaphors involving dancers and live music (Figure S5). Once the

models are established, the third act allows the Novice to use the models to explore a more complex phenomena while discussing the shortcomings of each model.

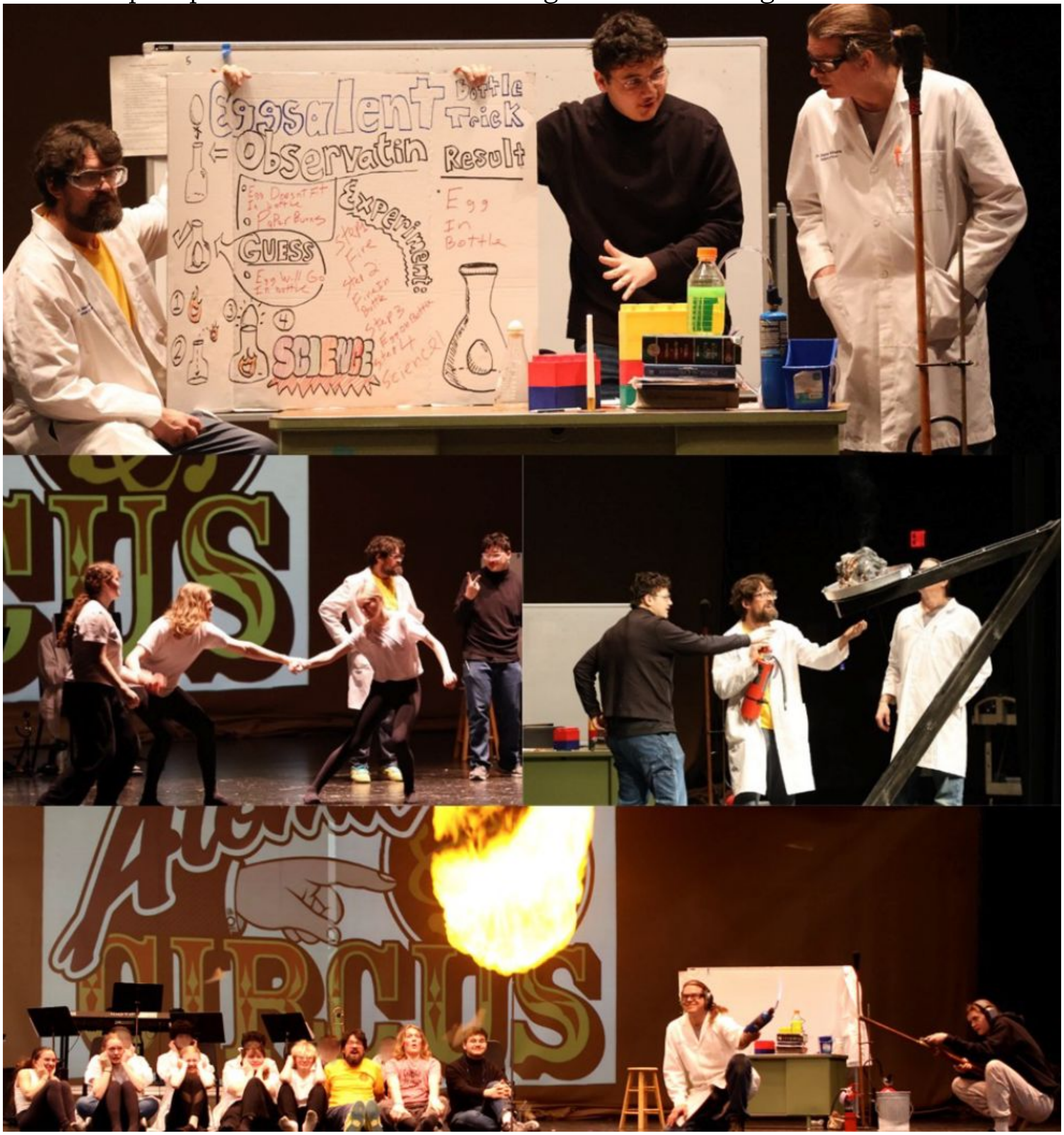

Figure S1. In The Atomic Circus Presents The Method To the Madness, the Novice, seeking help on a science fair project, discovers how hypothesis-driven science is conducted through a historic recounting of phlogiston theory. They use their new atomistic model to explain how the newly discovered oxygen combines with two hydrogens to make water.
Video S1: https://youtu.be/Km2jlpPLi8A?si=Fe1-CaqElkUBHTB0

Safety and Risk Management
All demonstrations are conducted by trained faculty under the Montana State University Billings Chemical Hygiene Plan and in compliance with institutional and state safety regulations. The performance team follows a formal pre-show safety briefing led by Dr. Dan Willems and he maintains a written equipment checklist for all transported materials. All chemical transport occurs in a Department of Transportation–compliant demonstration trailer. Audience members remain at safe distances, and only low-risk demonstrations involve volunteer participation (e.g., the "ice race"). All reactions using cryogenic or combustible materials are performed exclusively by faculty and supported by ventilation and protective shielding systems.

Table S1. List of Demonstrations and How They Are Performed

Demonstration	Description	Location / Controls
Butane bubbles	Ignited in open air by faculty; safety glasses and extinguisher on hand	On stage
Ice race	Volunteers melt ice using conduction	On stage (low-risk audience participation)
Liquid nitrogen smash	Shattering frozen objects to illustrate brittleness	On stage with safety shield
Barrel implosion	Sealed, steam-filled barrel cooled to create vacuum implosion	On stage, vented to exterior
Trash-can explosion	Hot water and liquid nitrogen expansion	On stage, steam vented outdoors
Gummy bear oxidation	Small-scale $KClO_3$ combustion in hooded cart	On stage, vented to exterior
Large gummy bear reaction	Scaled oxidation demonstration	Pre-recorded video segment
Hydrogen balloons	Variable H_2/O_2 ratios, loud reactions; earplugs provided	On stage
Diet Coke & Mentos vs. Elephant Toothpaste	Comparative physical vs. chemical reaction finale	On stage

Although modest in number, the deliberate pacing and scale of these demonstrations produce cumulative spectacle, reinforcing conceptual connections while maintaining safe practice.
Dr. Willems' Safety Officer character models risk assessment as part of scientific culture. Within the narrative, this figure enforces protective measures, narrates hazard awareness, and mediates between curiosity and caution—transforming safety from a backstage constraint into a visible element of scientific reasoning. This dramatization of safety as both procedural and ethical supports one of the show's core educational aims: to depict responsible experimentation as integral to authentic science.

Stage Design and Technical Setup

Performances take place on a proscenium stage. The curtain opens at the start of the show to reveal a set which includes an office desk, and chair, as well as a whiteboard-cart covered with hand-drawn formulas, sketches, and notes (upstage left). A single seat rolling student desk is situated on the stage left apron. Many fire extinguishers are visible next to the desk as well as downstage framing the stage, establishing both authenticity and comic exaggeration of laboratory safety culture. A three-piece band (guitar, bass, and drums) is situated upstage right. Downstage right is left open for dance sequences. A white scrim upstage functions as a projection surface for pre-recorded video segments and colored lighting effects.

Offstage, a mobile demonstration preparation station is situated in the stage left wing: this houses a two-burner propane stove for boiling water and a flammables cabinet for safe transport of reagents. In addition to this unit in the wings the backstage area features a kiddy pool filled with ice on a furniture dolly hidden behind the whiteboard along with all four balloons for the balloon demonstration. In addition to these items, a steel barrel sitting on a turkey-fryer burner fitted with a vent hood that exhausts through the rear stage door (backstage behind the scrim). Water is boiled in this barrel for the barrel implosion sequence. A decorated rolling demonstration lab bench sits in the stage right wing and is the primary vehicle for bringing demonstration on and off stage.

All performers use wireless ear-mounted microphones routed through a mixed PA system, while the three-piece rock band amplifies independently to preserve live dynamics. Two hanging decorations frame the stage visually, reinforcing the circus-like aesthetic. The minimalist but flexible configuration allows seamless movement between live demonstrations, dance sequences, and video cutaways without scene changes, supporting the principle of *theatrical metaphor*—physical simplicity enabling conceptual richness.

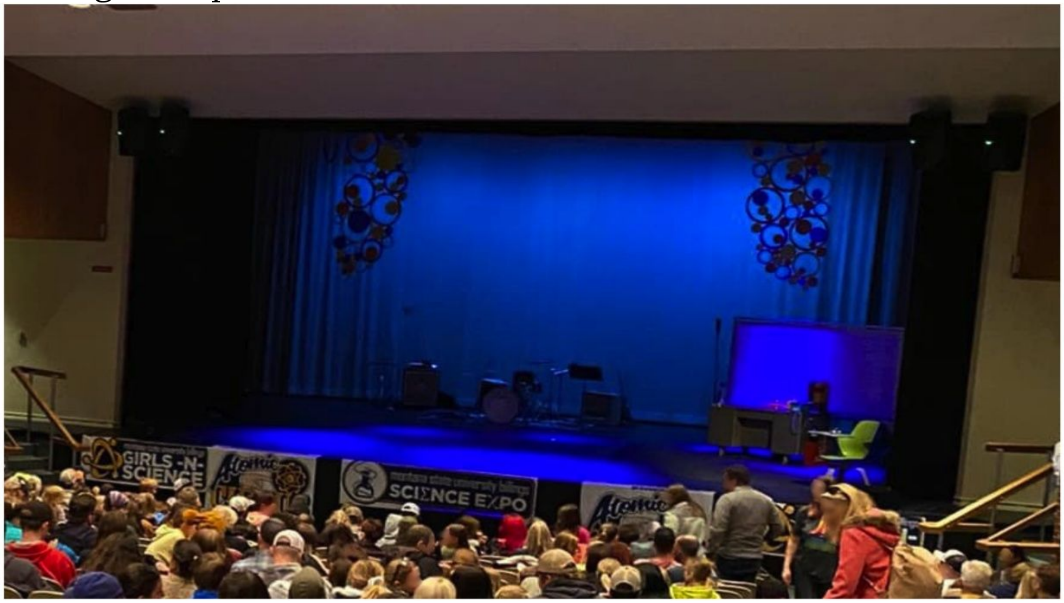

Figure S2: Note the open staging with room for dancing. The scrim offers cover for back stage preparation as well as a surface for projection.

Full Show Narrative Overview

Act I: The Search for a College Student

The show opens with the Expert, Safety Officer, and Lab Technician introducing themselves through a comic safety skit. The Expert lights propane bubbles in his hand, prompting the Safety Officer and Lab Tech to rush in with goggles, gloves, and a lab coat—an immediate cue to the show's blend of danger and discipline. Once "safely" attired, the Expert announces that today's lesson will explore chemistry *and* college life.

Spurred by sudden inspiration, the Expert decides that the audience needs a "real" college student to join the class. He exits dramatically through the theater doors, accompanied by hype music from the band. A live-feed mock-documentary—styled after classic wildlife or adventure programs—appears on the projection screen.

VIDEO S2. https://youtu.be/jhxhFfmUFGQ Finding the college student cutaway

The Expert narrates his search through the residence halls in the manner of a field naturalist, explaining habitats and behaviors. The sequence humorously parallels scientific observation while offering genuine information about majors and college years.

When the Expert "finds" a bewildered student, the conversation emphasizes that not knowing is part of learning—a motif that normalizes curiosity and confusion. The expert leads the student back into the theater as the doors burst open and the same student—the Novice—runs down the aisle wearing the identical costume from the video. The band resumes the hype theme as the audience chants "Stay! Stay! Stay!" to persuade the nervous newcomer to join the class.

Act II: Phase Change and the Molecular Circus

The second act begins once the Novice accepts the Expert's invitation to join the "class." Once committed to staying, the Novice asks, "What does a chemist even do?" prompting a short campus-interview montage projected onto the scrim. The clip ends with the Novice joking that chemists wear wizard hats and brew potions—just as the Safety Officer appears behind the Expert in a wizard cloak mixing dry-ice "potions." The Expert rebukes the stereotype and cues a fast-paced highlight reel showing real chemists at work. The scene concludes with a short lesson on atoms and the use of LEGO bricks as molecular models, establishing the show's recurring theme of *model-based reasoning* and leading directly into Act II.

Here, the focus shifts from the social world of college to the scientific world of matter, introducing *phase change* as the first major conceptual theme. This act exemplifies the narrative learning cycle—prediction, observation, reflection—woven with live demonstrations, dance, and music to model unseen molecular motion. The Expert opens with the *ice race*, a playful contest between a child volunteer and an adult. Each receives an ice cube, and the audience cheers as the competitors try to melt it fastest. The child secretly rubs their hands together, winning easily. The Novice, leading the chant "One, two, three… that's chemistry!", misidentifies the

result as a chemical change. The Expert interrupts with mock exasperation, explaining that melting is a *physical* change—ice and water are the same substance in different states. This lighthearted correction establishes a safe space for misconception and positions the Novice as a surrogate learner whose errors scaffold the audience's understanding.

To deepen the idea of physical change, the Expert turns to the whiteboard to sketch particle motion, but the lights fade and the music slows. The Novice dozes off, and the stage transforms into the Atomic-Level Circus, a dream sequence realized through lighting, dance, and live music. Dancers embody matter at the molecular level: in the "solid" section, they sway together in a fixed lattice; as tempo increases, they flow into a "liquid," gliding past one another; finally, they erupt into energetic, free-form leaps that fill the stage as "gas." The live band accentuates the transitions with rhythmic cues, using tempo as a metaphor for molecular energy. This scene enacts the show's guiding principle of theatrical metaphor, translating abstract kinetic theory into embodied experience.

Awakening from the dream, the Novice applies what they have "learned" to the next demonstration by predicting the existence of liquid nitrogen. The novice and expert become co-conspirators creating a mess of smashed frozen items. Eventually the Expert presents a balloon, asking what will happen when the balloon is immersed. Before the novice has time to guess they are seated and the Expert lecture starts. They fall asleep and wakeup again in the lend of the Atomic-Level Circus. a parachute dance follows: dancers move beneath a large collapsing parachute, slowing their motion as the fabric descends, dramatizing the relationship between temperature and molecular speed. The Novice awakens and correctly predicts the collapsing balloon (Figure S3).

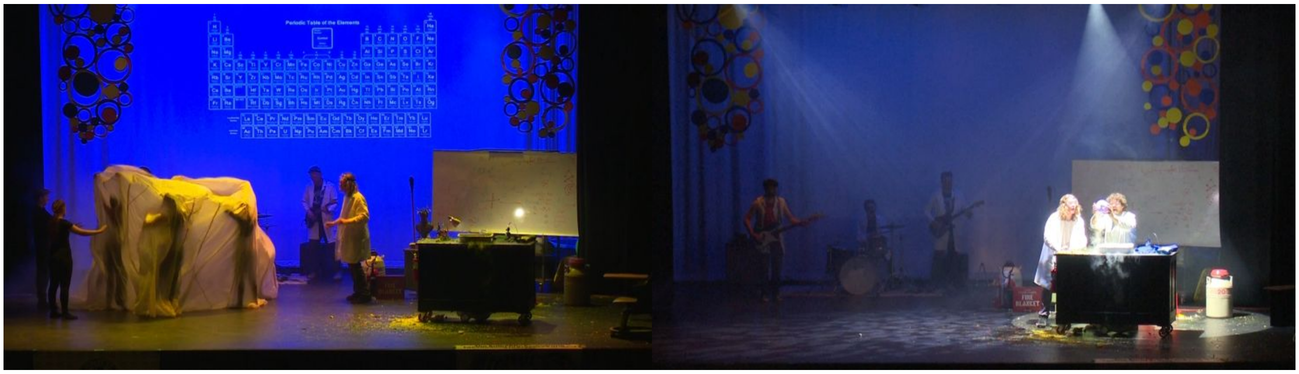

Figure S3. Parachute dance showing simulation of balloon in liquid nitrogen that motivates the predication followed by experimentation

The narrative cycle repeats and scales upward. The Lab Technician rolls out a kiddie pool filled with ice and a steel drum of steaming water. She claims that this can be done "bigger, and by bigger I mean better!" The Safety Officer warns of danger and begins the barrel implosion demonstration, the novice narrates connecting the small-scale balloon to a macroscopic system. As the heated drum is sealed and

cooled, it suddenly crumples with a thunderous implosion. The audience gasps, the band hits a triumphant chord, and the Novice exclaims his satisfaction with connecting the demonstrations. The Expert confirms that the same particle-motion principles apply, linking micro- and macroscopic phenomena through developmentally aligned modeling.

The physical change acts finale involves the now self-assured Novice correctly predicting that heating liquid nitrogen will produce rapid gas expansion. The Novice conspires with the Expert to sneak ping pong balls into a trash can filled with liquid nitrogen just as the safety officer throws in boiling water to create the gas, a playful act that defies the Safety Officer's disdain for messes. The predicted final explosion sends a cascade of ping pong balls flying across the stage, the audience erupts in cheers. This energetic finale marks the Novice's transformation from misconception to understanding they can now explain phase changes at the particle level using a model, sharing the process of conceptual growth with the audience.

Act III: Chemical Change and Transformation

The final act shifts from physical to chemical change, using spectacle to illustrate energy transformations at the molecular level. The segment begins with *sugar oxidation*: the Novice consumes an exaggerated spoonful of sugar, feigns discomfort, and asks how their body "burns" food for energy. The Expert explains that chemists can model this process by reacting sugar with oxygen. A small gummy bear demonstration follows under a portable fume hood, producing vivid light and sound. Interrupting, the Lab Tech, carrying a giant homemade 15 lbs gummy bear, declares, "We can do it better—and by better, I mean bigger!" They run off stage cutting to a live-feed video from their "off-site lab."

The pre-recorded sequence shows the fifteen-pound gummy bear being lowered into a vat of molten potassium chlorate as frantic music from the onstage band builds in synchrony. The reaction erupts into a fiery inferno; the feed glitches to static, and the Lab Tech re-enters the theater with a smoke-filled beaker and a singed lab coat, to the crowd's delight. The subsequent *sugar dance* translates combustion into motion: dancers portray molecules colliding, bonds breaking, and new products forming (linear CO_2 and bent H_2O). The choreography exemplifies *theatrical metaphor* and *multimodal modeling*, letting audiences visualize chemical reactions through embodied movement.

The next sequence illustrates *synthesis* via hydrogen-oxygen reactions. Four balloons are ignited in sequence—oxygen alone, hydrogen alone, a 1:1 mixture, and finally the stoichiometric 2:1 ratio. Each explosion grows louder, and the Novice graphs the intensity on the whiteboard, connecting auditory spectacle to reaction energetics. After the deafening 2:1 detonation (Figure S4), the band mock-storms offstage, only to be chased back by the Lab Tech pushing an enormous air-filled balloon. The cast dives for cover; the balloon bursts harmlessly, completing the comic learning cycle as the Novice explains why "nothing happened."

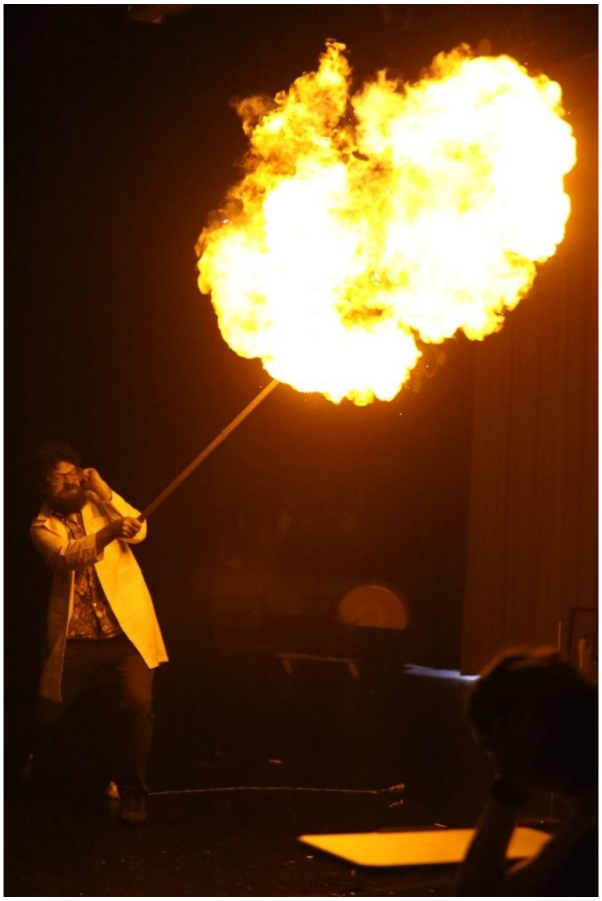
Figure S4. The final 2:1 H2:O2 balloon ignites the audience.

A *water dance* follows, where pairs of dancers attempt to lift symbolic oxygen atoms. Success occurs only when two hydrogens combine with one oxygen—embodying stoichiometric precision through motion. The Novice narrates the dance, reinforcing conceptual understanding while bridging humor and explanation.

The finale reprises the show's interactive spirit with a comparative demonstration of physical versus chemical reactions: Diet Coke + Mentos (physical) and Elephant Toothpaste (chemical). The audience is invited to decide which is which, a deliberately open-ended formative challenge that teachers often revisit in subsequent classroom discussions.

As the band reprises the opening theme, the Expert congratulates the Novice, who quips, "Wow—just another day in college, I guess." The ensemble leads the audience through a final call-and-response review of physical and chemical change. Exiting through the same doors they entered, the Novice waves goodbye—symbolically completing the learning journey from confusion to confident inquiry.

VIDEO S3: https://youtu.be/H76joSzLXUQ?si=nJs1-4uXIoNFaSz9

Table S2. Evidence-Based Design Principles Mapped to Atomic Circus Commitments for the 5th grade show

Theory-Informed Design Principle	Design Commitments	Examples in The Atomic Circus	Supporting Literature
**Age-Appropriate Conceptual Framing**	Content grounded in NGSS 5-PS1-4; emphasis on particle-level reasoning through visible analogies; avoid jargon, privilege models and analogies.	Novice eats sugar, talks with expert about sugar turning to water and carbon dioxide. Model it with gummy bear and potassium chlorate, and then explain via a dance.	Merritt & Krajcik (2013); Hadenfeldt et al. (2016); NGSS Lead States (2013)
**Narrative Storytelling**	Use characters as cognitive scaffolds (Novice = learner surrogate, Expert = mentor, Safety Officer = skeptical rationalist); maintain plot tension across three acts.	The Novice's misconceptions frame inquiry cycles; recurring chant "That's Chemistry!" becomes a metacognitive checkpoint; act transitions reinforce story coherence.	Bruner (1991); Fleer (2013); Engel et al. (2018); Klassen (2010)
**Theatrical Metaphor**	Employ music, dance, and projection as multimodal analogues for unseen processes; align tempo, lighting, and choreography with energy and molecular motion.	Dancers embody molecular behavior; music tempo tracks kinetic energy; projection overlays reinforce particle dynamics; lighting cues signal phase transitions.	Chemi & Kastberg (2015); Vieira & Morais (2021); Baljon et al. (2023)

These principles serve as transferable design heuristics rather than prescriptive frameworks. In subsequent productions (e.g., *The Atomic Circus Deconstructs the Scientific Method*), the same principles yield different commitments—narrative arcs shift, choreography changes medium, and conceptual focus aligns to new NGSS standards—demonstrating their adaptability.

Table S3 Continuation of Duggan et al. "Five Key Features of Studies" included in their review with the addition of The Atomic Circus.

**Study**	Study 1	Study 2	Study 3	Study 4	Study 5	**Our Study**
**Authors**	Roche et al. (2016)	DeKorver et al. (2017)	Fish et al. (2017)	Stojanovski (2017)	Karim and Roslan (2020)	Queen et al.
**Aim**	Provide an idea of a life as a scientist, engage the audience, use technology for audience interaction	Improve children's conceptual understanding of a molecular-level explanation for bouncing	Improve students' conceptual understanding of the physics of sound	Demonstrate the power of emotive, hands-on space outreach	Improve students' understanding of a range of physics concepts, and engage students in learning science	Improve understanding of physical and chemical change, while entertaining and connecting rural communities to higher ed and creative STEM modeling.
**Science Show Title (if present and topic(s))**	'Light': Developed for the UN-proclaimed 'International Year of Light and Light-based Technologies' celebration	The concept that the molecular interactions of an object give rise to its observable (macroscopic) characteristics	'Good Vibrations': the physics of sound	'Flight': topics relating to heat, forces, and pressure	'The Pressure Show': topics such as force and the Bernoulli Principle. 'The Fire Show': based on the fire triangle	The Atomic Circus Chemical and Physical Change
**Length**	-	30 mins (per format)	45 mins	20-25 mins (for schools)	Approx 45 mins (per show)	1 hour
**Format**	Basic demonstrations involving lasers and chemical reaction	Two formats: traditional format and FST format (both including the Happy and Sad Balls demonstration)	Demonstrations involving music, musical instruments (many local) and a range of multimedia	A small number of demonstrations interspersed with volunteers and audience participation	Eight to ten demonstrations per show. The students watched both shows on the same day	A three act play using chemistry demonstrations, actors, a live band, dancers, and visual projections.
**Setting**	Two festivals: (1) Electric Picnic, Ireland, (2) The British Science Festival, UK	Large science festival event at a university campus, USA	The Unizulu Science Centre, University of Zululand, South Africa	Primary and secondary schools, regional Australia	The Oil and Gas Discovery Centre (OGDC), Brune	University Theaters throughout Eastern Montana
**Audience**	Adults	Children (4–12 years) and their guardians	School students (14–15 years) from urban, township and rural schools	School students and the general public	School students (15-16 years old)	School shows for 5TH grade students, and public shows for families
**Presenter(s)**	Scientists with different backgrounds	Chemistry graduate students	The Director of the Science Centre	Science Communication Master's students	Experienced science show presenter and researcher	Chemistry professors, college age actors, an adult rock band, and teenage interpretive dance troupe.

Table S4. Extension of the Fusion Story Form: The Atomic Circus Story Form

Fusion Story Form Element	Learning Function (Kerby et al., 2018)	Atomic Circus Narrative Analog	How the Novice as Surrogate Learner Extends This Function
**Question / Prediction**	Elicits curiosity, elicits prior ideas	The Expert poses an unexplained demonstration or paradox ("Why did the barrel crush?")	The Novice voices naïve predictions, modeling student misconceptions; audience aligns with their curiosity.
**Evidence / Observation**	Provides sensory experience to challenge ideas	Demonstration performed live with theatrical flair; audience observes results with the Novice	The Novice reacts and articulates observations aloud, anchoring audience attention and focus; externalizes metacognition.
**New Ideas / Explanation**	Introduces new scientific constructs	Expert explains molecular model or rule; interpretive dancers illustrate microscopic phenomena	The Novice verbalizes comprehension, translating expert explanation into accessible language for the audience.
**Act-It-Out / Modeling**	Provides embodied particulate-level model	Dancers embody particles, energy transfer, or reactions	The Novice serves as bridge between macro observation and micro model; their awe and questions guide audience sensemaking.
**Revisit / Reflection**	Reinforces conceptual change, evaluates understanding	Novice applies model to new, scaled-up demo (e.g., barrel, gummy bear)	The Novice's transformation becomes a metaphor for community learning; the audience sees themselves in the learning journey.

Key Narrative Distinction

Unlike the Fusion Story Form's direct audience participation model, *The Atomic Circus* externalizes the audience's cognitive process through the Novice, a surrogate learner whose emotional and intellectual responses model curiosity, confusion, and eventual insight. This surrogate structure allows the performance to retain theatrical coherence for large audiences while preserving the learning-cycle logic (Prediction → Observation → Modeling → Reflection).

Evaluative Tools

Atomic Circus Show Pre-Survey

Q1 IRB Consent Form
- o Yes, I agree (1)
- o No (2)

Q2 Are you 18 or older?
- o Yes (1)
- o No (2)

Q3 Please enter a word and number combination (i.e. rock123) that you can remember to use when you fill out a survey after the show. This will allow us to match responses, but keep them anonymous. Thank you!

Q4 Have you seen the Atomic Circus before?
- o No (1)
- o Yes (2)

Q5 Have you seen other chemistry demonstration shows before?
- o No (1)
- o Yes (2)

Q6 How old are the people with you?

Q7 What is your race/ethnicity?
- o White alone (1)
- o Black or African American (2)
- o American Indian or Alaska Native (3)
- o Asian (4)
- o Hispanic or Latino (5)
- o 2 or more races (6)
- o other (7) ____

Q8 What is the highest level of education in your group attending the Show tonight?
- o Less than high school (1)
- o High School Degree or GED (2)
- o Some college (3)
- o 2 year degree (4)
- o 4 year degree (5)
- o Professional degree (6)
- o Doctorate (7)

Q9 Please respond with your agreement as a family to the following statements:

Strongly Agree (1) Somewhat agree (2) Neither agree nor disagree (3) Somewhat disagree (4) Strongly disagree (5)

We are interested in science (1)
We discuss science at home (2)
We seek out out-of-school opportunities to learn science (3)
Learning about science is important to our family (4)
Science is part of our daily lives (5)
We enjoy learning about science in school (6)
Our family works or aspires to use science in their careers (7)

Q10 How confident are you in knowing the difference between chemical and physical change?
o Definitely not (1)
o Probably not (2)
o Might or might not (3)
o Probably yes (4)
o Definitely yes (5)

Q12 When you put two substances together and a new substance is created that is a _______ change.
o Chemical (1)
o Physical (2)
o I don't know (3)

Q13 When substances change their state (i.e., gas to liquid, or liquid to solid) this is a _______ change.
o Chemical (1)
o Physical (2)
o I don't know (3)

Q14 When substances are heated do their molecules move faster or slower?
o Faster (1)
o Slower (2)
o I don't know (3)

Q15 Do molecules move faster in a gas than they do in a liquid?
o No (1)
o Yes (2)
o I don't know (3)

Q16 Why did you come to the Show today?

Atomic Circus Show Post-Survey

Q1 IRB Consent Form
o Yes, I agree (1)
o No (2)

Q2 Are you 18 or older?
o Yes (1)
o No (2)

Q3 If you took the pre-show survey, please share your word and number combination (i.e. rock123). Thank you!

Q4 I completed the pre-show survey
o No (1)
o Yes (2)

Q5 Have you seen the Atomic Circus before?
o No (1)
o Yes (2)

Q6 Have you seen other chemistry demonstration shows before?
o No (1)
o Yes (2)

Q7 How old are the people with you?

Q8 What is your race/ethnicity?
o White alone (1)
o Black or African American (2)
o American Indian or Alaska Native (3)
o Asian (4)
o Hispanic or Latino (5)
o 2 or more races (6)
o other (7) ____

Q9 What is the highest level of education in your group attending the Show tonight?
o Less than high school (1)
o High School Degree or GED (2)
o Some college (3)
o 2 year degree (4)
o 4 year degree (5)
o Professional degree (6)
o Doctorate (7)

Q10 Please respond with your agreement as a family to the following statements:
Strongly agree (1) Somewhat agree (2) Neither agree nor disagree (3) Somewhat disagree (4) Strongly disagree (5)
My family enjoyed the ACES Show (1)
This kind of out of school opportunity is important for increasing interest in science (2)

I learned something from the ACES Show (3)
The ACES Show related to my community (4)
The Show was well-produced (5)
I would attend a similar event in the future (i.e. family science event) (6)

Q11 How confident are you in knowing the difference between chemical and physical change?
o Definitely not (1)
o Probably not (2)
o Might or might not (3)
o Probably yes (4)
o Definitely yes (5)

Q12 When you put two substances together and a new substance is created that is a _______ change.
o Chemical (1)
o Physical (2)
o I don't know (3)

Q13 When substances change their state (i.e., gas to liquid, or liquid to solid) this is a _______ change.
o Chemical (1)
o Physical (2)
o I don't know (3)

Q14 When substances are heated do their molecules move faster or slower?
o Faster (1)
o Slower (2)
o I don't know (3)

Q15 Do molecules move faster in a gas than they do in a liquid?
o No (1)
o Yes (2)
o I don't know (3)

Q16 What did you learn at the show today?

Family Interview Protocol (post ACES show)

"Thank you for speaking with me regarding the ACES show. My name is xxx and I am conducting this interview as part of the evaluation about the impact of the Atomic Circus Show. We're trying to find out what people learned from the show, and also what they liked and didn't like about it. We're interviewing a few families and will bring all of the data together in a report for the organizers of the show. This interview should take 10-20 minutes and we'd like to offer you an Atomic Circus t-shirt for taking the time to speak with me today."

Have signed consent form for signature

1. Is it okay with you if I audio record this interview?
2. Can you tell me your name and age? (ask each participant)
3. What is your background/education level?
4. Race and ethnicity

"These first questions are for xxx (child) and then I've got some for xxx (adult)"

<u>Questions for children directly:</u>

1.
2. Do you think Cruz learned anything by the end of the show? What did he learn?
3. Would you want to be a scientist like Dr. Queen? Why or why not?
4. What will you tell someone about the show?
    a. Probe: What was your favorite part of the show?
5. Why did you attend today's show?
6. Do you have questions about anything you saw in the show?
7. Is there anything else you want to tell me about the show?

<u>Questions for the adult</u>

1. Why did you and your child attend the Show today?
2. What did you learn from the Show?
3. What did you think the messages of the Show were?
4. How well do you think the concepts in the show align with what your child is learning in school?
5. Were there specific aspects of the show that you think will spark conversations or discussions within your family (now/after the show or in the future…)?
6. What will you tell someone about the Show who didn't attend?
7. Have you been on the MSU-B campus before?
8. Is there anything else you would like to share?

References

1. Duggan, R.; Kranjc Horvat, A.; Schmeling, S.; van Sebille, E. The aims, contexts, evaluation and outcomes of science shows: a systematic review. *Int. J. Sci. Educ. Part B,* **2025**, 1-17.
2. Roche, J.; Cullen, R. J.; Ball, S. L. The educational opportunity of a modern science show. *Int. J. Sci. Soc.*, **2016**, *8*(3), 2.
3. DeKorver, B.K., Choi, M. and Towns, M. Exploration of a method to assess children's understandings of a phenomenon after viewing a demonstration show. *J. Chem. Educ.,* **2017**, 94(2), 149-156.
4. Fish, D., Allie, S., Pelaez, N., & Anderson, T. A cross-cultural comparison of high school students' responses to a science centre show on the physics of sound in South Africa. *Public Underst. Sci*, **2017**, 26(7), 806–814.https://doi.org/10.1177/096366251664272
5. Karim, N.; Roslan, R. The Impact of Interactive Science Shows on Student's Learning Achievement on Fire and Pressure Science Concept for 9th Grader in Brunei. *Jurnal Pendidikan IPA Indonesia*, **2020**, *9*(3), 294-308.
6. Stojanovski, L. Science show performance as a tool for space outreach: Lessons learned from the shell questacon science circus. In *68th International Astronautical Congress: Unlocking Imagination, Fostering Innovation and Strengthening Security, IAC 2017,*11435-11438. International Astronautical Federation, IAF, 2017.

7. Merritt, J; Krajcik, J. Learning progression developed to support students in building a particle model of matter. In *Concepts of matter in science education* **2013** (pp. 11-45). Dordrecht: Springer Netherlands.
8. Hadenfeldt, J. C; Neumann, K; Bernholt, S; Liu, X; Parchmann, I. Students' progression in understanding the matter concept. *J. Res. Sci. Teach.,* **2016**, 53(5), 683-708.
9. NGSS Lead States. Next Generation Science Standards: For States, By States. Washington, DC: The National Academies Press, 2013.
10. Bruner, J. The narrative construction of reality. *Critical inquiry*, **1991**, *18*(1), 1-21.
11. Fleer, M. Affective Imagination in Science Education: Determining the Emotional Nature of Scientific and Technological Learning of Young Children. *Res. Sci. Educ.,* **2013**, 43, 2085–2106.
12. Engel, A., Lucido, K. and Cook, K. Rethinking narrative: Leveraging storytelling for science learning. *Childhood Educ.,* **2018**, 94(6), 4-12.
13. Klassen, S. The relation of story structure to a model of conceptual change in science learning. *Sci. Educ.,* **2010**, 19(3), 305-317.
14. Chemi, T. and Kastberg, P. Education through theatre: Typologies of science theatre. *Applied Theatre Research,* **2015**, 3(1), pp.53-65.
15. Vieira, H; Morais, C. Bridging music and chemistry: A marching band analogy to teach kinetic-molecular theory. *J. Chem. Educ.,* **2021**, 99(2), 729-735.

16. Baljon, A. R. C; Alter, J. W; Bresciani Ludvik, M. J. Embodied Engagement with Scientific Concepts: An Exploration into Emergent Learning. *College Teach.,* **2023**, 71(1), 1–8.

17. Kerby, H. W.; DeKorver, B. K.; Cantor, J. Fusion story form: A novel, hybrid form of story that promotes and assesses concept learning. *Int. J. Sci. Educ.,* **2018**, *40*(14), 1774-1794.
